# Introducing post-discharge malaria chemoprevention (PMC) for management of severe anemia in Malawian children: a qualitative study of community health workers’ perceptions and motivation

**DOI:** 10.1186/s12913-018-3791-5

**Published:** 2018-12-19

**Authors:** Thandile Nkosi-Gondwe, Bjarne Robberstad, Björn Blomberg, Kamija S. Phiri, Siri Lange

**Affiliations:** 1 Centre for International Health, Department of Global Public Health and Primary Care, University of Bergen, P.O. Box 7804, 5020 Bergen, Norway; 2 School of Public Health & Family Medicine, College of Medicine, University of Malawi, Private Bag 360, Blantyre, Malawi; 3 Department of Clinical Science, University of Bergen, P.O. Box 7804, 5020 Bergen, Norway; 4 Norwegian National Advisory Unit on Tropical Infectious Diseases, Haukeland University Hospital, 5020 Bergen, Norway; 5 Department of Health Promotion and Development, University of Bergen, Postboks 7807, N-5020 Bergen, Norway; 6 Chr. Michelsen Institute, Jekteviksbakken 31, 5006 Bergen, Norway

**Keywords:** Anaemia, Malaria, Secondary prevention, Community health workers, Social perception, Malawi

## Abstract

**Background:**

Severe malarial anaemia is one of the leading causes of paediatric hospital admissions in Malawi. Post-discharge malaria chemoprevention (PMC) is the intermittent administration of full treatment courses of antimalarial to children recovering from severe anaemia and findings suggest that this intervention significantly reduces readmissions and deaths in these children. Community delivery of health interventions utilizing community health workers (CHWs) has been successful in some programmes and not very positive in others. In Malawi, there is an on-going cluster randomised trial that aims to find the optimum strategy for delivery of dihydroartemesinin-piperaquine (DHP) for PMC in children with severe anaemia. Our qualitative study aimed to explore the feasibility of utilizing CHWs also known as health surveillance assistants (HSAs) to remind caregivers to administer PMC medication in the existing Malawian health system.

**Methods:**

Between December 2016 and March 2018, 20 individual in-depth-interviews (IDIs) and 2 focus group discussions (FGDs) were conducted with 39 HSAs who had the responsibility of conducting home visits to remind caregivers of children who were prescribed PMC medication in the trial. All interviews were conducted in the local language, transcribed verbatim, and translated into English. The transcripts were uploaded to NVIVO 11 and analysed using the thematic framework analysis method.

**Results:**

Although intrinsic motivation was reportedly high, adherence to the required number of home visits was very poor with only 10 HSAs reporting full adherence. Positive factors for adherence were the knowledge and perception of the effectiveness of PMC and the recognition from the community as well as health system. Poor training, lack of supervision, high workload, as well as technical and structural difficulties; were reported barriers to adherence by the HSAs.

**Conclusions:**

Post-discharge malaria chemoprevention with DHP is perceived as a positive approach to manage children recovering from severe anaemia by HSAs in Malawi. However, adherence to home visit reminders was very poor and the involvement of HSAs in a scale up of this intervention may pose a challenge in the existing Malawian health system.

**Trial registration:**

ClinicalTrials.gov identifier NCT02721420. The trial was registered on 26 March 2016.

**Electronic supplementary material:**

The online version of this article (10.1186/s12913-018-3791-5) contains supplementary material, which is available to authorized users.

## Background

In sub- Saharan Africa, between 17 and 54% of malaria-attributable deaths are estimated to be due to severe anaemia [1]. Severe anaemia is a condition in which the number of red blood cells or their oxygen-carrying capacity is insufficient to meet physiological needs. Children with severe anaemia due to malaria are at risk of readmission and death within 6 months after discharge from hospital [2, 3]. Post-discharge malaria chemoprevention (PMC) is the intermittent administration of full treatment courses of antimalarial to children recovering from severe anaemia. Findings from studies in Malawi and other African countries suggest that PMC to children in the post-discharge period significantly reduces hospital re-admissions and deaths after discharge from hospital [2–19]. Although PMC is a relatively simple intervention, its implementation requires a feasible and acceptable delivery strategy [9, 20–25].

Community health workers (CHWs) and related cadres play a role in prevention, case management and health promotion delivery in many poor countries [26–28]. Delivering community-based programs using community health workers has been shown to be effective in achieving high coverage, improved access, and in reducing maternal, neonatal and child mortality in some resource-poor settings [9, 20–22, 29–38]. However, the global health literature has also identified a number of challenges related to the role of CHWs in different community-based interventions [38–42].

In the Malawian health care system, CHWs are known as Health Surveillance Assistants (HSAs). The HSAs are the lowest cadre in the Malawi health system and the Ministry of Health (MoH) pays them a monthly salary. In the villages, they work together with community leaders in providing some basic health and environmental health services to the community and it is estimated that each is expected to serve approximately 1000 people in a catchment area [43]. Their role is important, since it is estimated that only 54% of the Malawian population resides within 5 km radius of a health facility and even patients who do reach a health facility encounter a shortage of health workers [29]. Furthermore, the proportion of the population residing within 5 km of a health facility equipped to provide treatment of severe anaemia is far lower. HSAs have been reported to create a link between the health facility and the community and they have contributed to the delivery of health services in rural areas [44, 45].

The literature refers to a number of factors that influence CHW activity, motivation and continuation on the job [46–54]. Motivation can be defined as the “willingness to exert and maintain an effort towards goals” and is regarded to develop in individuals as a result of the interaction between individual, organizational and cultural determinants [55]. In Malawi, HSA motivation has been found to be driven by community interactions and opportunities for increased responsibility [29]. Other reported contributing factors to sustained motivation and job satisfaction are altruism, need for community respect and recognition, supportive supervision and monetary and non-monetary incentives [45]. However, the literature also shows that HSAs are not always conducting the activities that are expected from them. For example, a recent study of caregivers’ perceptions of delivery of PMC showed that caregivers reported that HSAs were unreliable and mothers do not prefer them as a means to receive reminders [56].

The main objective of this study is to assess the feasibility of involving HSAs in the scaling up of PMC into the health system by exploring perceptions, experiences and motivation of the HSAs who were involved in an on-going trial in Malawi [57]. The study will contribute to the existing literature on the role of CHWs in low-income settings, and particularly to a better understanding of the interrelationship between motivation and structural factors for scaling up an intervention such as PMC.

## Methods

### Study place

The study was conducted in Zomba District, which is located in the southern region of the Republic of Malawi and has a population of approximately 883,200. Public health services in Malawi are provided mainly at village health posts, health centres and district and central hospitals. There are 32 health facilities in Zomba district and a total of 630 HSAs working for the Ministry of Health (MoH).

### The design of the trial

This study is part of a trial that aims to find the optimum strategy for delivery of PMC. A total of 375 children aged less than 5 years are given an antimalarial medication; dihydroartemisinin-piperaquine (DP), over a period of 3 months during the recovery from severe anaemia. The delivery of the medication is divided into five different arms, of which two are facility-based and three community-based and the protocol for the trial has been described elsewhere [57]. This study focuses on one of the community-based study arms where the study medication is given to the caregiver upon discharge from the hospital, and she/he is instructed to give them to the child at two, six and ten weeks after discharge for 3 days at a time. The dates are noted on the child’s health card.

The PMC trial recruits children from the Zomba central hospital (ZCH), which is the referral hospital for all neighbouring health centres and districts. On the day when the child is discharged, the caregiver is linked to the HSA in their catchment area. The HSA responsible for the catchment area receives their contact details and are informed about the participant and the location of their home. In the case that the HSA is not available, he/she is contacted by phone, or by receiving a physical visit, with information about the participant’s details, location and treatment procedures. One or two days prior to the scheduled date of drug administration, the HSA was expected to receive a short message service (SMS) reminder on his/her phone to go to the child’s home to remind the caregiver to administer the study medication. The HSAs did not receive any other reminders or memory aids during the course of follow up and they were not paid specifically for this work because it was considered to be part of their regular duties. However, they received a lunch allowance during the initial training.

Prior to commencement of the trial, community engagement and briefing about the trial was conducted with all the 7 Traditional authorities (TAs) and their group village headmen. In Malawi, TAs are key community leaders. In these meetings, information about the trial objectives and methods were discussed and the leaders were expected to disseminate this information in their communities. In addition, 608 HSAs received a short training about PMC as an intervention for management of children with severe anaemia. The training included informing them about their role in the project whereby they were requested to visit the child and remind the caretakers to administer the study medication to the child. The HSAs were not shown the actual study medication during the training, and there was no formal assessment of knowledge attained.

### Study participants and sampling

A total of 78 children were randomized to receive the intervention in the arm utilizing HSAs, and 60 HSAs participated in the trial. Of these, 21 were not interviewed since they had either relocated, were unreachable by phone, or had a child participating at the time of the study and could not be involved in the interviews to avoid influencing outcome measures of the trial. The remaining 39 HSAs were invited to participate in this study. In December 2016 we conducted 20 individual in-depth interviews (IDIs) with HSAs, and later, in March 2018, we conducted two focus group discussions (FGDs) with 19 HSAs at designated health centres. While saturation to a large degree was achieved with the first 20 IDIs, the two FGDs gave us an opportunity explore further the findings that had been identified in the initial analysis of the IDIs. The choice of the additional 2 FGDs was made in order to explore different experiences between adherent (FGD1) and non- adherent (FGD2) HSAs. Inclusion was not based on age or gender, but rather by caregiver’s report at the exit visit of the main trial.

### Data collection

The semi-structured interview technique was used utilising a guide with set topics, and an open-ended approach, which allowed the informant to speak without interruption. This was best suited for this study because we aimed to uncover the perceptions and understandings of PMC from the interviewees’ point of view [58]. All interviews were conducted in the local language; Chichewa, by three experienced and independent research assistants. The interview guides were formulated in English and translated into Chichewa and piloted with the local nurses to ensure that the information gathered truly reflected the study objectives and ensured that probes were captured precisely (Additional files 1 and 2). Furthermore, all research assistants and the first author were Malawians, who speak fluent Chichewa. Since the first author is the medical researcher responsible for conducting the trial, she took part only in the interviews with HSAs that she did not have prior contact with to reduce the likelihood of biased responses. The information gathered was supplemented by her experiences and observations throughout the trial and field notes made during the interviews. All interviews were digitally recorded, transcribed verbatim and translated into English by the research assistants. Permission to conduct the trial was obtained from ZCH and Zomba DHO prior to commencement and written consent was obtained from all participants.

### Data entry and analysis

The first author went through all the transcripts, observation notes and audio files for accuracy and reliability, and read each transcript carefully to familiarise herself with the data. The last author reviewed all the FGD transcripts. Transcripts were loaded into NVIVO 11 and a coding system was developed based on the research objectives and the analytical framework of motivation. The coding system was expanded during the coding process to capture emerging themes outside of the original study objectives. The first author coded each IDI and FGD and the last author reviewed the coding frame. Through this process the coding was refined and both agreed on the final themes. Thematic framework analysis was used because it is an appropriate, rigorous and systematic method for undertaking qualitative analysis [59, 60].

## Results

### Characteristics of participants

A total of seven women and thirteen men were interviewed in the IDIs. The HSA ages ranged from 27 to 45 years and the length of service as HSAs was between seven and 24 years. A total of thirteen HSAs served a health centre that was categorised as rural and seven were from the urban or semi-urban health centres. FGD 1 comprised of three women and six men and seven women and three men in FGD 2 respectively. The ages ranged between 32 and 45 years in FGD 1 and 27 and 51 in FGD 2. In FGD 1, the range of service as HSAs was nine to twenty years and eight and seventeen years in FGD 2. All the discussants in FGD 1 reported to a rural health centre while six out of the ten reported to a rural health centre in FGD2. The range of duration of each IDI and FGD interviews was between 62 to 104 min. A majority of all had completed secondary school and attained a Malawi school certificate of education (MSCE), while four possessed a junior certificate of education (JCE), which is attained after completing 2 years of secondary school. The HSAs reported that their catchment populations ranged between 599 and 33,000. A summary of the characteristics of the IDI and FDG participants is provided below in Tables 1 and 2 respectively.

**Table 1:** Characteristics of HSAs who participated in IDIs

HSA ID	Gender	Age	Location category	Population size	Length of service	No. Of visits	Level of adherence
HSAs with self- reported adherence (*N* = 12)							
ID1-HSA-01	Male	32	Urban	2219	9	1	Partially adherent
IDI-HSA-03	Male	40	Semi-urban	894	7	3^a^	Fully adherent
IDI-HSA-04	Female	37	Rural	3077	10	1	Partially adherent
IDI-HSA-05	Female	45	Urban	4050	24	3	Fully adherent
IDI-HSA-06	Female	36	Semi-rural	Unsure	9	2	Partially adherent
IDI-HSA-07	Male	35	Rural	3090	9	1	Partially adherent
IDI-HSA-09	Female	39	Semi-urban	1139	8	2	Partially adherent
IDI-HSA-10	Male	30	Rural	3299	10	1	Partially adherent
IDI-HSA-11	Male	39	Remote	2360	14	2	Partially adherent
IDI-HSA-14	Female	30	Semi-rural	2202	9	1	Partially adherent
IDI-HSA-17	Male	42	Semi-rural	4500	17	3	Fully adherent
IDI-HSA-18	Male	27	Urban	1160	9	1	Partially adherent
HSAs with self-reported non-adherence (*N* = 8)							
IDI-HSA-02	Female	27	Urban	884	9	0	Not adherent
IDI-HSA-08	Female	42	Urban	599	21	0	Not adherent
IDI-HSA-12	Male	33	Semi-rural	1276	9	0	Not adherent
IDI-HSA-13	Male	32	Rural	1356	8	0	Not adherent
IDI-HSA-15	Male	32	Rural	4000	7	0	Not adherent
IDI-HSA-16	Male	38	Remote rural	NA	10	0	Not adherent
IDI-HSA-19	Male	36	Remote	940	9	0	Not adherent
IDI-HSA-20	Male	40	Remote	3100	9	0	Not adherent

^a^This HSA had 2 children during the course of the study and he was fully adherent to both

**Table 2:** Characteristics of HSAs participating in two FGDs

HSA ID	Gender	Age	Location	Population	Length of service	Number of visits
FGD 1: HSAs with full or partial-adherence as reported by the caregiver						
FGD-HSA-01	F	32	Rural	982	10	2
FGD-HSA-02	M	42	Rural	3840	18	2
FGD-HSA-03	F	39	Remote	1796	20	3
FGD-HSA-04	M	35	Rural	1139	10	4
FGD-HSA-05	M	35	Rural	9808	12	3
FGD-HSA-06	M	38	Rural	2098	11	3
FGD-HSA-07	F	35	Rural	2876	10	2
FGD-HSA-08	M	34	Rural	3614	9	3
FGD-HSA-09	M	32	Rural	9300	10	2
FGD 2: HSAs with non-adherence as reported by the caregiver						
FGD-HSA-10	F	27	Rural	2070	10	0
FGD-HSA-11	F	35	Semi urban	890	8	0
FGD-HSA-12	F	45	Semi urban	883	10	0
FGD-HSA-13	M	29	Urban	1896	10	1
FGD-HSA-14	F	45	Urban	1982	10	0
FGD-HSA-15	M	38	Remote	1740	10	3
FGD-HSA-16	F	31	Semi urban	2339	10	0
FGD-HSA-17	F	32	Semi urban	1038	10	0
FGD-HSA-18	M	39	Remote	1935	10	0
FGD-HSA-19	F	51	Rural	2040	17	3

### Adherence

HSAs were required to visit the child’s home three times, at two, six and ten weeks after discharge from hospital. Information on adherence to home visits was collected at the exit interview with caregivers as part of the trial, and during IDIs and FGDs with HSAs. In FGD 1, all the nine HSAs were reported as adherent by caregivers. Out of the 9 HSAs, 3 of them report to have conducted at least one visit; 2 reported full adherence and 1 had partial adherence. The ten HSAs in FDG 2, in contrast, were reported to be non-adherent by caregivers. However, three of them claimed that they had made one or more visits to the child. In the IDIs, twelve HSAs reported adherence but two differed from what the caregiver reported. There may be several reasons reported for this discrepancy. Firstly, the HSA may have visited the child when the caregiver who was interviewed for the exit interview was not at home. Second, the caregivers or HSAs may not have reported correctly. Third, there may have been a social desirability effect; HSAs may have wanted to over-report their adherence. In this paper, we refer to the HSAs self-reported adherence. Tables 1 and 2 also summarize adherence level by HSA.

We define adherence level by the number of home visits the HSAs conducted. Those that conducted all three home visits are termed “adherent”, those who made at least one visit are termed “partially adherent” and those that did not conduct any home visit are termed “non-adherent”. Only ten HSAs, or about one in four, carried out all the three required home visits. Of the remaining three quarters of HSAs, fifteen were non-adherent and fourteen were partially adherent. We did not find any clear association between HSAs characteristics (age, sex, population size) and adherence, but we note that adherence was slightly higher among HSAs in rural communities (30%) compared to those who were based in urban areas (15%). Additionally, we noted that male HSAs had the highest reported adherence compared to female HSAs; but the sample was too small to make any generalization. Table 3 below summarizes the adherence levels among HSAs categorized by location.

**Table 3:** Adherence to PMC home visits by location

Location	Adherence	Total (%)
Rural (*N* = 26)	≥3	8 (30)
	1–2	10 (39)
	0	8 (30)
Urban (*N* = 13)	≥3	2 (15)
	1–2	4 (30)
	0	7 (54)

In the following, we map out factors that appear to have influenced HSAs’ adherence. We have categorized these factors into three main categories: Professional factors, structural factors, and community factors. For each of them, we describe a number of enabling and motivating factors reported by the HSAs. We also describe the barriers and demotivating factors that HSAs identified for each of these categories, which reportedly discouraged adherence.

### Professional factors

The belief that PMC is important and useful, gaining new knowledge, and opportunities to conduct other tasks were reported as motivating factors, while the lack of incentives was reported as a demotivating factor by some HSAs.

#### The belief that PMC is important and useful

All HSAs, regardless of adherence, expressed that malaria is a serious disease and is the most common cause of death among children in their communities. Regarding knowledge of severe anaemia, the HSAs explained that it is a complication of malaria and most described those with severe anaemia as “having white hands” and that this condition frequently leads to death.

All HSAs, including the non-adherent, perceived PMC as an important and beneficial intervention that will save the lives of many children. In addition, HSAs that were adherent to at least one visit expressed that the children who took the medication did not get sick from malaria again:

*This study has helped - the child’s frequent malaria problems have ended. Even when the child doesn’t look sick, the drugs are still given, to completely kill the malaria parasite. This would benefit the nation. (FGD1, male, 34 yrs, adherent)*

#### Gaining new knowledge

Of the 39 participants, seven had not been part of the initial training. Of these seven, two had received information on the day that the child was taken home by the research team, while the other five had heard about it from their fellow HSAs. None of the HSAs had prior knowledge about the fact that there is medication that can be given to children in order to protect them from getting malaria. This knowledge was appreciated and considered novel by all HSAs regardless of adherence, but it was more important to the HSAs who were adherent. They said this knowledge gave them confidence in the medication and gave them a sense of pride to know something that even superiors at their facilities didn’t know:

*We have gained knowledge and the kids have been helped (FGD 1, male, 34 yrs, adherent)*

They also said this knowledge was beneficial to other people than the children in the trial since they spread this information to other community members as well as health workers at their facilities. However, more than half of all the HSAs reported that the information received on the day of the initial training was too limited. They also argued that follow-up trainings would have been a big motivator for them because they would have gained refreshed knowledge and up-to-date information.

*Everyone knew about this study, but the information was not clearly laid out to us. As my colleague has already pointed out, my plea is that next time there is a meeting, the information should be well delivered to us, and we should have enough details. The training should have been for a few days, or even a week. In my case, it turned out that the client had more information than me so it is sort of embarrassing. (FGD 1, male, 35 yrs, adherent)*

#### Recognition by government or NGOs

Most of the adherent HSAs reported that the fact that they were recognised and considered to take part in reminding caretakers is a sign of respect to them and recognition of their importance in the health system. They expressed that they are often not involved during inception of similar projects and yet they are the ones who provide health care within the community:

*As HSAs, we are supposed to be part of this because the people are ours, we live with them in the community, and we know them in and out. When they (hospital staff) come here they just register and leave. But we know their homes, how it is, what’s there, their habits, maybe their eating habits, we visit their homes and we know. But then the problem is when things like these come; they involve clinicians, nurses and so when it gets hard, that’s when they involve the HSAs at the end. (IDI-05, female, 45 yrs Adherent)*

However, many of the non-adherent HSAs were not happy about being involved in projects organized by NGOs and research institutions. They felt that they were being “used” since there is no personal benefit to them:

*To say the truth, I was supposed to deliver the message. But the benefit is not there for me because the study was just done with me, they have just used me. So it means much benefit is gained by those getting the support of drugs, and those doing the study. I was just supposed to be doing my job as I do. (IDI-12, male, 33 yrs, non-adherent)*

#### Genuine love for the job, sense of obligation and altruism

Intrinsic motivating factors are factors that make an individual to carry out tasks without outside influence. Many participants reported that being an HSA was a calling and that it was their responsibility to ensure good health in their communities. Altruism can be described as the opposite of selfishness and it’s the inert need to do good. Among the HSAs that conducted at least one visit this was an emerging theme. Several reported that the love for the job and the love for their community enabled them to help the children get better and that they had saved lives:

*Of course, I like it because our role is to save people’s lives. So, after you have followed up and you find that they are getting better, you feel that you had done the job on your own, but when the person is neglecting it and then they die, you feel like you haven’t done your job, like you are the one who has killed them. (IDI-01, male, 32 yrs, partially adherent)*

Many expressed that it is a calling and a privilege to serve the communities that they are part of, but some were vocal that their motivation was also linked to financial rewards.

#### No personal rewards

Very few of the non-adherent HSAs reported that provision of additional incentives (other than those provided by the government as part of their job description), would improve their willingness to visit the children in their homes. Many HSAs however, both adherent and non-adherent, argued that they should get more training (which also means a per diem), and two of the HSAs wanted bicycles as a way to be motivated to conduct the task. When asked what kind of incentive that would be more motivating, the majority preferred incentives in the form of additional training with financial allowances rather than being paid per visit to the child, as this is not practical.

*Just as my fellow HSA has said, getting these trainings will motivate us to do the work. We shouldn’t be given allowances every time we visit a child but rather when we attend training. (FGD 2, female, 45 yrs, non-adherent)*

### Structural factors

Structural factors are factors that are closely linked to the practicalities of the work tasks themselves. Many of the HSAs who were adherent reported that the task was easy, and/or that they knew the participant. However, we also identified a number of structural factors that were barriers to adherence. These barriers are factors that were reported by HSAs who were not adherent or partially adherent. We have categorized these factors into workload, inadequate training and lack of supervision, difficulties related to text messages, and lack of transport. Although the scope of our study was particularly expressed in the context of PMC activities, most of the reported structural barriers are true for the HSA’s current daily work.

#### Ease of the task and opportunity to conduct other activities that are within job description

The majority of the HSAs who were adherent or partially adherent reported that the task was easy, uncomplicated and not time-consuming:

*It was simple; I didn’t spend much time, since it was just to go and remind them and I would come back, and continue with my work. (IDI-10, male, 30 yrs, partially adherent)*

They described that they went to the child’s home and simply ensured that child was given medication. Most of them said that the entire visit took less than 10 min and was even easier than other tasks they do on a daily basis:

*Since our job is mostly about visiting our people in the community, we are able to pass through the client’s home whilst attending to other activities. (FGD 1, male, 35 yrs. adherent)*

Moreover, more than half of them had found that either the caregiver had already administered the medication or had already planned to do so. Although the task was easy, they felt that their visit was very important because it made a positive impact on the caregivers and in turn motivated them to remember to give medication to their children. A few also added that these visits gave them the opportunity to conduct other activities such as giving health counseling, nutritional assessments and family planning counseling to the caregivers and therefore they were able to incorporate it into their schedule:

*It is not something that is done frequently, or eats up most of our time. When we visit the client we also encourage them to follow healthy methods of life such as building a toilet. Through these visits a bond is created between the clients and us. (FGD 1, male, 42 yrs, partially adherent)*

#### Knowing the participant, distances and transport

Adherence was highest among HSAs who knew the location of the family that they were to visit. HSAs in this group had either been informed or taken to the home by the research team or they received a message from their supervisor. Four of them made the effort to find the location prior to the scheduled visits when they received a message. Among these HSAs, all of them mentioned that as a result of knowing the location or the family, it was much easier to conduct the visits and they felt an obligation to make the home visits.

Similarly, most of those that were non-adherent, reported that they did not know the child or the location when they received the message. Nearly all of them said they did not make the effort thereafter to find the location. However, two of the non-adherent HSAs knew the study participants’ home, but despite this they did not conduct the visits because they were informed to wait for SMSs, which they didn’t receive:

*Yes, I was given the client name and was told that I will receive a phone call but I didn’t. I did not follow up the child even though I knew him, because I was waiting for more details. I will receive a message of when to go, but I did not receive any, hence I didn’t visit the child. (FGD 2, male, 39 yrs, non-adherent)*

However, most of those that eventually become aware of the child and their home cited that the distance to the participant was too far to go at such short notice. They felt that they were not able to go a day or two after the specific date because it would be too late.

Transport was reported to be a challenge also by some of the adherent HSAs. One HSA who conducted all three visits mentioned that the child lived very far but he was only able to visit her because he decided to schedule clinics in a nearby village around the dates the PMC medication was due:

*Actually, I have an outreach site close to her home. And the dates she was given for the dosage were corresponding to those when I was having my outreach nearby. Hence, once I was done with my activities, I would go visit the child. (FDG 2, male, 38 yrs, adherent)*

He said this could only be achieved because he knew the schedule. Another HSA who also conducted all the visits reported that she would have liked to have a bicycle because the child’s home was far away:

*It was far. I had to go around a mountain to get to her place. (FGD 2, female 51 yrs, adherent)*

#### Workload, PMC not part of HSAs regular work

Although all HSAs concurred that serving their community plays a major role in their motivation to work, most of the HSAs that were not adherent reported that at the time they were supposed to go to visit a child for PMC they were already expected to perform many other tasks, including tasks meant to be done by nurses and clinicians. One participant said he was aware about the child under his care, but he “simply forgot” to go because he had so much to do. Another HSA also said that PMC is not on his job description, just like another on-going project whereby they are requested to go and give vitamin supplements. They all agreed that it is very difficult to introduce new tasks for children aged less than 5 years, since they already entail a lot of activities such as vaccinations and vitamin supplementation that are scheduled on different dates.

*We were all given work that does not even concern us but we just do it. When actually, it’s not in line with our job description. It is not there. So, they pile a lot of work on you, yet the pay itself does not match the given jobs. (IDI-13, male, 32 yrs, non-adherent)*

Although HSAs who were adherent reported that PMC visits were relatively simple they similarly expressed that it increased workload, which is particularly challenging since PMC reminders are time sensitive and therefore more difficult to combine with other duties. Two of the HSAs had the responsibility to remind more than one caregiver and both of them were adherent. One of them mentioned that although he did not find it difficult, visiting more than one child during the same time period in the case of an upscale of PMC would be too much work on top of the existing assigned tasks.

#### Inadequate training and lack of supervision

The majority of the HSAs were dissatisfied with the training that they received for the PMC study. They said that the training prior to the study was too short with too much information. In addition, the medication instructions were not very clear. This was a reported barrier even among those who were adherent. They complained that there was no follow-up training following the initial one. As a result, they had forgotten most of the content and some even said they thought the project was finished. They reported that they only followed instructions as per the short message reminder they received during the trial and not during the initial training.

A major complaint from HSAs was the fact that they saw the medicine for the first time only with the caregiver, because the medicine was not shown during the training:

*The work is not demanding. My only problem was that I did not know the medicine that was administered to the child. None the less it was simple. (FGD 2, male, 29 yr, partially adherent)*

Not having seen the medication was disappointing to the HSAs, because they did not want to look incompetent in front of the caregivers. They feared losing the confidence of the caretaker because as HSAs they are considered highly qualified and are expected to have all the answers. In this case, when they showed ignorance about the medication the caregiver was unsure whether to give the medication or not. This was expressed by both HSAs who made the visit, as well as those that didn’t:

*You could have informed us more about the medicine during the briefing. We had no idea how they look like; hence it would be a surprise to see that kind of medicine when we visit the client. We are like doctors to the clients and if they see our ignorance about the medicine, every guardian will question whether they should really give the dosage to their children. (FGD 2, female, 31 yrs, non-adherent)*

Most of the HSAs reported that their supervisors were not involved in any way during the intervention. The responsibility solely lied on the responsible HSA to ensure that home visits were conducted according to plan. They also complained that this supervision was lacking also from the research team. Most said it should be the supervisor who has to ensure that the work plan of each HSA reflects all activities assigned but none of the supervisors offered any guidance. One said the supervisor also had limited information and as such he was not able to go and visit the child:

*I had to check with my supervisor for details. My supervisor did not have any concrete information about it as well; though he attended the meeting he said the training wasn’t clear enough. (FGD 2, male, 39 yrs, non-adherent)*

One HSA who did not conduct any home visit said she was away on maternity leave at the time the child was discharged and felt that had the supervisors been involved, another HSA could have taken over reminding the child on her behalf.

#### Difficulties related to text messages

Two or three days prior to drug administration, HSAs were to receive text messages to their personal phone from the study team with the following information translated into the local language: “Go and remind (child’s name) to give medicine from tomorrow for three days”. The majority of the HSAs, both those who were adherent and those who were not, reported that they did not receive all the monthly text message reminders. For some of the non-adherent, this was reported as one of the reasons why they had not visited the child:

*I did not know the date that the medicine was supposed to be taken because the date was known by the project team so they were supposed to call me that the child is supposed to take the medicine tomorrow. And then I was supposed to go and see the child in the morning and tell them that the child is supposed to take medicine at this particular time. How would I go when I do not know the dates that the child is supposed to take the medicine? (IDI-6, female, 36, partially adherent)*

Four non-adhering HSAs reported that they had been informed during the training to only visit the child when they received a text message, and because they did not receive it they did not conduct a single visit:

*I couldn’t do anything because we were told to wait for the phone messages. At least if they called us, we would have monitored the child. (FGD 2,male, 39 yrs, non-adherent)*

The few who received the text message said that having received it was helpful, although not required. They instead relied on a schedule they developed after finding out the dosing schedule documented in the child’s health book. It was reported that the content of the SMS was adequate and clear. Only one HSA expressed that the information on the reminder was not clear and therefore he did not know which child to visit.

Others reported difficulties with the text message was that the timing of the text message was very inconvenient because it came at a time when they had either concluded their daily activities or were away. One HSA who conducted only one visit reported that he received the text message once and that was the time when he was able to go and remind the caretaker. Another HSA decided to go even if he hadn’t received the message:

*The greatest problem I encountered was waiting to receive a message of reminder. I did not receive the message so I would go on my own to visit the client. (FGD, male, 38 yrs, adherent)*

Another pointed out that he is not reachable by phone every time due to unreliable network coverage or inability to have the phone charged all the time since he lives in a remote village:

*Maybe sometimes you have been calling me but you couldn’t get through, it is because my phone doesn’t get through, since the phone is a phone by name (meaning the phone is unreliable). (*IDI-07, male, 35 yrs., partially adherent)

The majority of the participants expressed that more focus should be put on providing enough information including dose schedule to HSAs because having this information made it possible for them to incorporate it into their scheduled work plans and also to conduct the home visits even when text messages were missing. Similarly, HSAs who were non-adherent reported that if they had known the dosing schedule beforehand they would have been able to plan ahead, but otherwise they didn’t know what was going on:

*The first time I visited the child, I took the child’s health card to check out the dates on which the drugs should be taken. So, I would remind the guardian on such given date. I was motivated even more when I would find the child has already been given the drugs. I did not receive any message of reminder. The first time I visited the client, I just got the dates on which I should be reminding them. (FGD 2, male, 29 yrs, partially adherent)*

Overall, HSAs reported that the SMS reminders were not necessary due to their unreliability.

### Community factors

In addition to professional and structural factors, factors that were related to the HSAs relationship with the community, such as their wish to maintain community trust was important for adherence.

#### Maintaining community trust

Another theme that was emerging was that all HSAs, regardless of adherence level, argued that they had a strong link with their villages and community members. They expressed that the recognition by their communities as “doctors” gives them pride. As mentioned before, we noted that adherence was highest among HSAs from rural locations of which most live within the communities they serve. They report that there is a level of trust and confidence that their communities hold for them and it is important to maintain it as they conduct their daily tasks:

*To my understanding I have been able to help the clients and in return creating a bond with them. They will have a good perception about me and trust me when need arises. I am appreciated for the job I have done. (FGD 2, female, 51 yrs, adherent)*

For them, going to the child’s homes for this project was no different and maintaining the confidence motivated them to go. Although those who were non-adherent talked in a similar way, they did not see this as a major determinant of motivation for them to go.

#### Caregivers see the benefit of PMC, but neighbours may be sceptical

When asked about community reception and attitudes towards the intervention, most adherent HSAs reported that caregivers of children taking PMC were generally very receptive and had a good understanding and information about the study medication, although some parents did not understand the idea of giving medication to children who are not sick or have recovered.

More than half of the HSAs reported that the caretakers had a better understanding of PMC than themselves and all fully adherent HSAs reported that they were certain that all prescribed doses were given to the child because caregivers were determined to improve their child’s heath.

However, some of the HSAs reported that generally, there was a lack of information and understanding of PMC by community members who were not directly involved. Two HSAs specifically mentioned that as much as the caretakers whose children were receiving PMC were happy to give medication to their children, some of the neighbours were curious and suspicious:

*To some people who don’t know about this study, they were very suspicious about it. They would ask why the frequent visits to that house and why health officers keep coming there. Whenever I sat outside with the mother, some people would pretend to come greet me while they just want to eavesdrop on what’s happening. I asked the lady if we could go inside, as this was irritating. The mother would feel uncomfortable about it but I had to remind her the importance of this study to her and the child. So, the challenge I had was the other people had a bad perception about the study. (FGD 1, male, 42 yrs, partially adherent)*

As a result of this, two of the HSAs reported that caregivers feared raising unwanted suspicion among their fellow community members as to why they were being repeatedly visited by the HSA, which would result in being stigmatized. One of them explained that the caregiver avoided being at home when the HSA was supposed to visit and he did not find her at home on two consecutive visits. The other HSA reported the same concerns but he simply encouraged the mother to continue giving her child medication:

*Maybe the people may be wondering, “Why are they coming to see our children?” maybe they don’t want you to come because of what people say. But yet in the end they may accept it. (IDI-07, male, 35 yrs partially adherent)*

Some of the HSAs also reported that there are some cultural and social beliefs that exist in the communities such as that a chronic illness in a child is often attributed to witchcraft or karma and that there are no medications for it. A few of the HSAs also mentioned that some community members believed that the medicine is given to the children so that they should be infertile. However, this was not presented as a major factor that affected adherence to conduct home visits but rather an observation that may result in caregivers not being adherent.

*They just say “you are giving these things to the children with an aim of destroying them so that they should not have children in future. They should not do this and that.” This is what most people ask and so you have to convince them that it is not like that. (IDI-04, female, 37 yrs,partially adherent)*

When presented with the idea that caregivers could be in full charge of the medication, some argued that HSAs should be central to this and that it would be better if the medication were kept by them and not by the caretakers to guarantee proper storage and administration:

*If an HSA would be taught on how to dispense these drugs that they receive at the central hospital, they can be left at the facility, when the follow up date comes the HSA will take the drugs, go and give it to the mother and also see how the child is doing. (IDI-10, male 30 yrs, partially adherent)*

Four of the adherent HSAs however, expressed that caregivers are capable of remembering to give medication and may not need to be reminded by HSAs because some of the caregivers had already given the study medication at the time when the HSA was visiting. The findings of this study have been summarized in Table 4 below.

**Table 4:** Summary of findings

	Enabling and motivation factors	Barriers and demotivating factors
Professional factors	- The belief that PMC is useful - Gaining new knowledge - Maintain recognition from the government and NGOs - Genuine love for the job, altruism, sense of obligation	- Sense of PMC not being part of regular work duties - No personal reward - No provision of earmarked financial incentives or other incentives like air time, bicycle or additional training with allowances
Structural factors	- Ease of the task - Short distance to child’s home - Knowing participant location and family - Being able to combine with other duties in the same area	- High workload - Not knowing participant or family location - Long distance to child’s home, lack of transport - Inadequate information and lack of supervision - No refresher trainings - Not receiving SMS/No phone - Had not seen the study medication, fear of losing care takers’ respect because of this
Community factors	- To maintain community trust and respect as ‘doctors’ - “Love for the community” - Care takers see the benefit for their child - Being informed by the care taker about the dosing schedule	- Curiousness and suspicion from neighbours - Caretakers may fear stigmatisation - Fear that the study medicine may be harmful

## Discussion

In this study we aimed to explore whether utilizing HSAs can be a feasible, practical and acceptable strategy in the delivery of PMC. We found that only one in four HSAs who were trained to incorporate PMC reminders into their normal duties were adherent to all three visits. However, despite this poor level of adherence, the great majority of the HSAs report that they are intrinsically motivated to remind caregivers to administer dihydroartemesinin-piperaquine to children in their community without any additional financial incentives.

We found that the main motivation for HSAs in this intervention came from their altruism and the recognition that they play a major role in provision of health services to the community and the appreciation they get from the caregivers. Kadzandira et al. and Kok et al. reported that HSAs are a link between the community and the health system in Malawi where their major strengths reported for motivation are team spirit, cooperation, knowledge of local culture, language and environment [44, 45]. The HSAs’ need to attain and maintain the trust of the community gives them a sense of duty and responsibility because they have developed lasting bonds with the people within the community [51, 55, 61]. This was further outlined in our study in that they want to maintain that level of power by insisting that they need to be an integral part of this intervention. However, when it came to conducting the duties assigned for this intervention this motivation was not demonstrated.

In addition, the experience of actually seeing the impact of their work and benefits of the intervention play a major role on motivation, which was similarly reported by Winn et al. and Gilroy et al. in other malaria programmes [62, 63]. We found that most of the HSAs do not expect additional financial incentives to conduct home visits since similar visits are part of their normal duties anyway. Some studies have reported that financial incentives may represent a source of sustained motivation, while others have observed that they can negatively influence motivation [43, 55, 64]. On the other hand, a number of the HSAs stressed that lack of refresher training (with allowances) reduced their motivation to make PMC visits. This is consistent with findings reported by Mpembeni et al. and Prytherch et al. in studies from different community-based interventions [55, 64].

This suggests that some form of reward either financial or non-financial for the extra work is needed to drive their extrinsic motivation, as they genuinely want to do well in their communities. Financial incentives in the form of lunch allowances during trainings would have the added benefit of gaining new knowledge.

### If intrinsic motivation is high then why was there such poor adherence?

Although awareness of the burden and impact of malaria and severe anaemia was high among the HSAs, the question is why there was poor adherence to an intervention that could potentially minimise morbidity and mortality in the communities? In line with other studies, our study found that the factors that influence HSAs motivation to carry out the PMC intervention activities are multilayered [48, 49]. Professional factors allow for good intentions to carry out the duties because most are interpersonal but structural and community factors are a hindrance to them. Some of the non-adherent and partly adherent participants in this study referred to a high workload as one reason why they had not conducted the visits. High workload has been reported as a barrier for CHWs also in other studies [43–45]. However, most said the task was simple, so perhaps in the event of a scale up there would not be the amount of resistance as demonstrated here. Moreover, in the event of an upscale, PMC will most probably be incorporated into the regular work description of the HSAs, and will not be seen as an additional task.

We have identified some significant structural barriers that contributed to poor adherence: difficulties with the text messages which meant that HSAs were not informed when to visit the child, inability to locate the child, and transportation challenges. The poor functioning of the SMS reminders suggests that one should not design a system that relies on the use of this technology in the event of an upscale in Malawi.

All HSAs received similar training, but there are other factors that come into play because with the same training some HSAs were adherent to all the required visits. Limited information and knowledge about the intervention meant that the HSAs did not have a proper understanding of their role and expectations. There was no follow up supervision because we didn’t want to affect adherence, which is a major outcome of the main trial. Many studies have shown similar findings, that training improves outcomes because without retraining acquired skills and knowledge are lost over time [43, 61, 63, 65–69]. The finding that the majority of the HSAs felt that they needed follow-up trainings, suggests in the event of an upscale, that resources should be placed into conducting more frequent training as a way of providing additional knowledge and feedback [66].

Strengthening supportive supervision has also been shown to improve effectiveness of utilising CHWs in similar contexts. Good supervision has been shown to be an effective way to boost team spirit and improve the work environment through a continuing dialogue in similar contexts [64, 66, 68–70]. Clarity of chains of command and accountability are reported as sources of extrinsic motivation, since one fears rebuke and loss of professional respect. When it comes to anchoring PMC reminders, it would be better if there were existing chains of command. However, introducing such lines of command in the current study, might have had an undesired impact on the main trial outcomes because this would falsely represent an existing operational system for supervision of HSAs.

Reminding caregivers to give PMC medication is reportedly simple and requires minimal resources in the cases when the child lives nearby, but most of the challenges were related to identifying the child and incorporating that into their routine scheduled work plans. Locating the child was a challenge for some. In the cases that the HSAs were available, they were driven to the child’s home by the research team. However, in the event of a scale-up, HSAs will not be driven to the child’s home. Therefore, to address this, establishing and strengthening existing supportive supervision is important.

HSAs in this study report having strong relationships with their community who depend on them for guidance and community health needs. Caregivers in the trial preferred to use reminders documented in the child’s health book rather than the HSAs reminding them. They report that their HSAs were unreliable and often unavailable [56]. However, other reports have shown this to be opposite in that HSAs have close ties with the community and is an important driver for carrying out their duties [20, 49]. Interestingly, the HSAs themselves report that most of the times the caregivers were capable of administering the medicine to their children without reminders from them. However, the main concern and reason for being part of for this would be to reduce misuse in terms of administration of the medicine to another child and poor storage conditions due to the limited community understanding. They therefore believe that they should be an integral part of this and work hand in hand with the caregiver.

### Study limitations

Our study was done in the context of a clinical trial, which may have affected our findings in several ways. First, some HSAs may have felt that since PMC was an add-on to their regular work which they were not given any extra compensation for, they were not “obliged” to be adherent, and an integration with regular chain of command in a national scale up might potentially improve adherence. Second, and pulling in the opposite direction, in the event of a national upscale, HSAs will not be transported to the child’s home and adherence may therefore be even lower than what we found. The feasibility of sending a customized SMS with the specific child’s name may also not be sustainable in the long run, given the constraints facing the health system. Finally, the results have probably been affected by the social desirability effect whereby the HSAs might have over-reported their enthusiasm and willingness to conduct the visits.

## Conclusion

This study has explored the feasibility of delivering the PMC intervention by utilizing HSAs to remind caretakers in the delivery of PMC as a strategy of management of severe anaemia during the post discharge period. We found that HSAs in Malawi perceive PMC with dihydroartemesinin-piperaquine as an important intervention and that they are intrinsically motivated to conduct home visits to remind caregivers to administer the medication to their children. Nevertheless, adherence to the visits was poor.

Difficulties with the SMS reminders, not knowing the location of the child and/or long distances, and lack of information about the medicine currently limits motivation, gives practical challenges and reduces adherence. In the event of a national scale up of the intervention, the responsibility for delivering PMC should not be anchored with HSAs, but HSAs can provide an additional support to care takers as long as this is included in their job description, and the supervision system is strengthened. Provision of regular trainings, which provide a lunch allowance, may provide sustained motivation and provide the opportunity to enhance their knowledge. However, addressing most of these challenges will be difficult in the current health system. Unless additional resources are invested, utilizing HSAs to remind caregivers as a delivery strategy for PMC may not be feasible.

## Additional files

Additional file 1: HSA interview guide; Interview guide for in-depth inter-views with health surveillance assistants in the post discharge malaria chemoprevention study. (DOCX 29.0 kb)

Additional file 2: HSA _FGD interview guide; Interview guide for focus group discussions with health surveillance assistants in the post discharge malaria chemoprevention study. (DOCX 28.0 kb)

## Abbreviations

CHW: Community health worker
DHMT: District health management team
DHO: District health office
DP: Dihydroartemesinin-piperaquine
FGD: Focus Group discussions
HSA: Health surveillance assistant
IDI: In-depth-interviews
IPTc: Intermittent preventive therapy in children
IPTi: Intermittent preventive therapy in infants
IPTp: Intermittent preventive therapy in pregnancy
IPTpd: Intermittent preventive therapy post-discharge
PMC: Post-discharge malaria chemoprevention
SMC: Seasonal malaria chemoprevention
SMS: Short message service
WHO: World Health organization
ZCH: Zomba Central hospital
