# Supplementary material for: Introducing post-discharge malaria chemoprevention (PMC) for management of severe anemia in Malawian children: a qualitative study of community health workers’ perceptions and motivation
Source: BMC Health Serv Res. 2018 Dec 19;18:984. doi: 10.1186/s12913-018-3791-5 (PMC6299958; doi:10.1186/s12913-018-3791-5)
Supplement: Supplementary file 1 — HSA interview guide; Interview guide for in-depth inter-views with health surveillance assistants in the post discharge malaria chemoprevention study. (DOCX 29.0 kb) [file 12913_2018_3791_MOESM1_ESM.docx]

IDI GUIDEVersion 1.1_final10/19/2016

**INTERVIEW GUIDE FOR IN-DEPTH INTERVIEWS WITH HEALTH SURVEILLANCE ASSISTANTS IN THE POST DISCHARGE MALARIA CHEMOPREVENTION STUDY**

Introduction

Personal information

Name: Age:

Occupation (any other work than HSA?):

Marital status:

If married/co-habitant, occupation of partner:

Religion:

Highest qualification:

Basic HSA training duration:

Other trainings:

Length of service as HSA:

Length of service in this catchment area:

Size of catchment area, number of villages, population and distance from residence:

Total number of village health volunteers:

Reporting health center:

Consenting process (Refer to informed consent form)

1. **Background and motivation**
   1. Did you grow up in this area? (if not, when did you move here and why?)
   2. You say that you have been an HSA for x years (see above). What did you do before you became a HSA?
   3. Can you tell us why you decided to become a HSA? (if mention love for people/wish to help the community, explore this in depth. If he/she doesn’t mention it, leave it)
   4. Did you consider other kinds of work before becoming a HSA? (What was the person’s dream in life? Lack of other work opportunities in this area?)
2. **Roles of HSAs in the community**
   1. What are your role as a HSA in this community?

(Probe for each role mentioned, time spent on each)

- 1. Are these roles in line with your job description according to Ministry of Health?
  2. What challenges do you face for each role mentioned?
  3. Have you ever been part of a project that required you to visit individual community members? If yes, which project?
  4. Can you please mention all the projects that you have been involved with through NGOs/donors (all other actors than the Ministry of Health)? For each project: Please explain your role, when they took place, who funded, for how long, if any allowances were paid, and what you think of the project – did you like it or not? Why/why not?)

1. **Perceptions about malaria and anemia**
   1. What do you know about Malaria? Causes, complications and outcomes?
   2. If anemia not mentiond: What do you know about anemia? What is it caused by?
   3. Do you see many cases of anemia due to malaria in children? Approximately how many cases do you see in your catchment area in a year?
   4. How are these children managed at community level and primary health center level?
   5. What happens when these children have been referred to a tertiary hospital?
   6. What happens when these children have been discharged from hospital?
   7. What information is provided to you by the mother or your supervisor about these children?
   8. Do you have any thoughts on how children with severe anemia due to malaria can be managed after discharge from hospital?
2. **Knowledge about the PMC study**
   1. What do you know about the PMC study?
   2. What information was given to you regarding this study and its interventions?
   3. Were you satisfied with the training/information you received?
   4. How did you feel about being given this extra task? Why happy/unhappy with being given this task?
3. **Experiences with PMC**
   1. Did you have a child in the PMC study whom you were responsible for?
   2. Did you know this child’s family from before? If yes, how well? (family, friends, close friends, neighbours, just know by face, know them by name but don’t know them well?)
   3. Did you receive a SMS to remind you to go and remind the guardian to give study drugs? (Probe number of times and content of SMS)
   4. How did you fit PMC activities into you workload?
   5. Did you go visit the child? (Probe number of times, time period)
   6. What activities did you do when you went to visit the child? (Probe about information given to mother, examinations)
   7. What challenges and constraints did you face conducting PMC activities?
   8. Did you benefit from the PMC trial in any way?
   9. How receptive were the caretakers towards you when you went to their homes?
4. **Perceptions about PMC as an intervention for management of severe anemia**
   1. What do you think about PMC?
   2. Is it a useful intervention for management of children with severe anemia post discharge?
   3. Do you think HSAs should take part in PMC if it were to become national policy? Why/why not?
   4. What challenges do you think will be there if PMC is to be implemented nationally?
   5. What other interventions do you know for management of these children?
   6. What are the perceptions of the community about PMC? (Probe for specific group views).
5. **Job satisfaction**
   1. After (all) these years as a HSA, are you happy with your decision to become a HSA? Why/why not?
   2. What aspects of your work do you like the most? (probe for examples)
   3. Is there something you don’t like? (fear of infections, long distances)
   4. Have you ever considered quitting your work as a HSA? Why/why not?
   5. Do you think that you will be working as a HSA ten years from now?
   6. In your view, what are the most important personal qualities a HSA should possess? (Try to make them say what comes to their mind, then probe for medical knowledge, intelligence, following guidelines/instructions, kindness/interpersonal skills, empathy/love for people, what is more important?)
6. **Social status of HSAs**
   1. In your view, are HSAs respected by the community? Why/why not? Examples? Depends on the individual HSA?
   2. Are HSAs respected by higher level health workers? Why/why not? Examples?
   3. Are HSAs respected by the government? Why/why not? Examples?
   4. What is your monthly take home salary (after tax, but excluding loans, if any)
   5. What do you feel about your salary? (sufficient, a little too low, way to low?)
   6. Is your salary normally paid on time? (if not, how often delayed)
   7. Do you have any other income? (probe for other work, farming, poultry/dairy, informal trade etc, and if his/her role as a HSA is useful for ‘business’/net working)
   8. If married/cohabitant: Is your HSA salary higher or lower than your partner’s income?
   9. Do you have children? If yes, would you advise them to become a HSA?
   10. Do you have any other role in this community? (Through local politics, church/mosque, CSO etc). If yes, do you get any allowances/income from this?
7. **Closure**
   1. Do you have any questions to us, or anything you would like to add?
   2. Thank you very much for your time.

NB: Optional for those who did not conduct the visits. According to our data you did not visit the mother and child. Please know that we are not judging, we would just like to know why, so that we can understand the work of HSAs better”.
