# Supplementary material for: Introducing post-discharge malaria chemoprevention (PMC) for management of severe anemia in Malawian children: a qualitative study of community health workers’ perceptions and motivation
Source: BMC Health Serv Res. 2018 Dec 19;18:984. doi: 10.1186/s12913-018-3791-5 (PMC6299958; doi:10.1186/s12913-018-3791-5)
Supplement: Supplementary file 2 — HSA _FGD interview guide; Interview guide for focus group discussions with health surveillance assistants in the post discharge malaria chemoprevention study. (DOCX 28.0 kb) [file 12913_2018_3791_MOESM2_ESM.docx]

FGD GUIDEVersion 1.0_final02/03/2018

**INTERVIEW GUIDE FOR FOCUS GROUP DISCUSSIONS WITH HEALTH SURVEILLANCE ASSISTANTS IN THE POST DISCHARGE MALARIA CHEMOPREVENTION STUDY**

Introduction

Personal information (For each participant)

Names: Age:

Marital status:

If married/co-habitant, occupation of partner:

Religion:

Highest qualification:

Basic HSA training duration:

Other trainings:

Length of service as HSA:

Length of service in this catchment area:

Size of catchment area, number of villages, population and distance from residence:

Total number of village health volunteers:

Reporting health center:

Consenting process (Refer to informed consent form)

1. **Knowledge about the PMC study**
   1. What do you know about the PMC study?
   2. What information was given to you regarding this study and its interventions?
   3. Were you satisfied with the training/information you received?
   4. How did you feel about being given this extra task? Why happy/unhappy with being given this task?
2. **Experiences with PMC**
   1. Did you have a child in the PMC study whom you were responsible for?
   2. Did you know this child’s family from before? If yes, how well? (family, friends, close friends, neighbours, just know by face, know them by name but don’t know them well?)
   3. Did you receive a SMS to remind you to go and remind the guardian to give study drugs? (Probe number of times and content of SMS)
   4. How did you fit PMC activities into you workload?
   5. Did you go visit the child? (Probe number of times, time period)
   6. What activities did you do when you went to visit the child? (Probe about information given to mother, examinations)
   7. What challenges and constraints did you face conducting PMC activities?
   8. Did you benefit from the PMC trial in any way?
   9. How receptive were the caretakers towards you when you went to their homes?
3. **Perceptions about PMC as an intervention for management of severe anemia**
   1. What do you think about PMC?
   2. Is it a useful intervention for management of children with severe anemia post discharge?
   3. Do you think HSAs should take part in PMC if it were to become national policy? Why/why not?
   4. What challenges do you think will be there if PMC is to be implemented nationally?
   5. What other interventions do you know for management of these children?
   6. What are the perceptions of the community about PMC? (Probe for specific group views).
4. **Job satisfaction**
   1. After (all) these years as a HSA, are you happy with your decision to become a HSA? Why/why not?
   2. What aspects of your work do you like the most? (probe for examples)
   3. Is there something you don’t like? (fear of infections, long distances)
   4. Have you ever considered quitting your work as a HSA? Why/why not?
   5. Do you think that you will be working as a HSA ten years from now?
   6. In your view, what are the most important personal qualities a HSA should possess? (Try to make them say what comes to their mind, then probe for medical knowledge, intelligence, following guidelines/instructions, kindness/interpersonal skills, empathy/love for people, what is more important?)
5. **Social status of HSAs**
   1. In your view, does the community respect HSAs? Why/why not? Examples? Depends on the individual HSA?
   2. Are HSAs respected by higher-level health workers? Why/why not? Examples?
   3. Does the government respect HSAs? Why/why not? Examples?
   4. What is your monthly take home salary (after tax, but excluding loans, if any)
   5. What do you feel about your salary? (Sufficient, a little too low, way to low?)
   6. Is your salary normally paid on time? (If not, how often delayed)
   7. Do you have any other income? (Probe for other work, farming, poultry/dairy, informal trade etc, and if his/her role as a HSA is useful for ‘business’/net working)
   8. If married/cohabitant: Is your HSA salary higher or lower than your partner’s income?
   9. Do you have children? If yes, would you advise them to become a HSA?
   10. Do you have any other role in this community? (Through local politics, church/mosque, CSO etc). If yes, do you get any allowances/income from this?
6. Some of you said that you felt a bit demotivated since there were no personal benefits. How do you feel about this?
7. What factors made it not possible/possible for you to visit? Probe for
   1. Distance to child home
   2. Receiving SMS
   3. Information received
   4. Training received
   5. Not knowing dosing schedule before hand
   6. Negative community reception
   7. The ease of job was demoralising’
8. For those who did not conduct the visits according to caregiver’s report: “According to our data you did not visit the mother and child. Please know that we are not judging, we would just like to know why, so that we can understand the work of HSAs better”.
9. **Closure**
   1. Do you have any questions to us, or anything you would like to add?
   2. Thank you very much for your time.
